# A new rauisuchid (Archosauria, Pseudosuchia) from the Upper Triassic (Norian) of New Mexico increases the diversity and temporal range of the clade

**DOI:** 10.7717/peerj.2336

**Published:** 2016-09-06

**Authors:** Emily J. Lessner, Michelle R. Stocker, Nathan D. Smith, Alan H. Turner, Randall B. Irmis, Sterling J. Nesbitt

**Affiliations:** 1 Department of Geosciences, Virginia Polytechnic Institute and State University (Virginia Tech), Blacksburg, VA, United States; 2 Department of Biological Sciences, Virginia Polytechnic Institute and State University (Virginia Tech), Blacksburg, VA, United States; 3 The Dinosaur Institute, Natural History Museum of Los Angeles County, Los Angeles, CA, United States; 4 Department of Anatomical Sciences, Stony Brook University, Stony Brook, NY, United States; 5 Natural History Museum of Utah, University of Utah, Salt Lake City, UT, United States; 6 Department of Geology and Geophysics, University of Utah, Salt Lake City, UT, United States

**Keywords:** *Teratosaurus*, *Postosuchus*, Rauisuchidae, Hayden Quarry

## Abstract

Rauisuchids are large (2–6 m in length), carnivorous, and quadrupedal pseudosuchian archosaurs closely related to crocodylomorphs. Though geographically widespread, fossils of this clade are relatively rare in Late Triassic assemblages. The middle Norian (∼212 Ma) Hayden Quarry of northern New Mexico, USA, in the Petrified Forest Member of the Chinle Formation, has yielded isolated postcranial elements and associated skull elements of a new species of rauisuchid. *Vivaron haydeni* gen. et. sp. nov. is diagnosed by the presence of two posteriorly directed prongs at the posterior end of the maxilla for articulation with the jugal. The holotype maxilla and referred elements are similar to those of the rauisuchid *Postosuchus kirkpatricki* from the southwestern United States, but *V. haydeni* shares several maxillary apomorphies (e.g., a distinct dropoff to the antorbital fossa that is not a ridge, a straight ventral margin, and a well defined dental groove) with the rauisuchid *Teratosaurus suevicus* from the Norian of Germany. Despite their geographic separation, this morphological evidence implies a close phylogenetic relationship between *V. haydeni* and *T. suevicus*. The morphology preserved in the new Hayden Quarry rauisuchid *V. haydeni* supports previously proposed and new synapomorphies for nodes within Rauisuchidae. The discovery of *Vivaron haydeni* reveals an increased range of morphological disparity for rauisuchids from the low-paleolatitude Chinle Formation and a clear biogeographic connection with high paleolatitude Pangea.

## Introduction

There is much confusion in the phylogeny and taxonomy of the Triassic ‘rauisuchians.’ That group typically references large-bodied, mostly carnivorous pseudosuchian archosaurs that clearly are not aetosaurs, phytosaurs, ornithosuchids, or crocodylomorphs (Gower, 2000; Nesbitt et al., 2013), but establishing the relationships of this assortment of large, quadrupedal, Triassic predators has been challenging. Groups traditionally referred to Rauisuchidae and Rauisuchia are paraphyletic (Nesbitt, 2011, but see Brusatte et al., 2010), but comprise a number of potentially monophyletic subgroups including Prestosuchidae, Poposauroidea, and a much more restricted Rauisuchidae (Brusatte et al., 2010; Nesbitt, 2011). Currently, there is consensus regarding ingroup relationships within some of the smaller clades, but the relationships between the subgroups and to other pseudosuchians remain contentious (Brusatte et al., 2010; Nesbitt, 2011; Lautenschlager & Rauhut, 2015).

Despite the overall disagreement between their respective phylogenetic hypotheses, the analyses of Brusatte et al. (2010) and Nesbitt (2011) recovered a similar taxonomic makeup of Rauisuchidae, which includes *Polonosuchus silesiacus*, *Postosuchus kirkpatricki*, and *Rauisuchus tiradentes. Teratosaurus suevicus, Tikisuchus romeri*, and *Postosuchus alisonae* were hypothesized to be additional possible members of this group (Brusatte et al., 2010; Lautenschlager & Desojo, 2011; Nesbitt et al., 2013).

Excavations from 2004 to 2015 at the Hayden Quarry at Ghost Ranch, New Mexico, USA have recovered a number of new rauisuchid skeletal elements. Previously, all Late Triassic rauisuchid fossils from Texas, Arizona, and New Mexico were referred to *Postosuchus kirkpatricki*, including those from the nearby Canjilon Quarry at Ghost Ranch (Long & Murry, 1995). The discovery of a rauisuchid clearly distinguishable from *Postosuchus* provides reason to reevaluate all previously referred rauisuchid material from the southwestern United States. Here, we describe these skeletal elements from the Hayden Quarry and erect a new taxon *Vivaron haydeni* gen. et. sp. nov. Our comparisons are either through first-hand examination of specimens and/or digital reconstructions of computed tomographic (CT) data from relevant rauisuchid specimens. Our analyses reveal that rauisuchids occupied a large biogeographic range with a wide latitudinal distribution over Pangea during the Carnian and Norian (Benton, 1986; Nesbitt et al., 2013).

## Materials and Methods

The rock matrix of the Hayden Quarry ranges from mudstone/siltstone to intraformational sandstone/conglomerate (Irmis et al., 2011). B-72 was used in the field as a consolidant and removed in preparation using water and acetone. The bone is well preserved, black, and is accompanied by charcoal. All material was mechanically prepared (by EJL, SJN, and other volunteers) by pinvise and air scribe, using B-72, B-76, and cyanoacrylate adhesives. The holotype maxilla (GR 263) was CT scanned on September 26, 2014 at the Virginia-Maryland Regional College of Veterinary Medicine in a Toshiba Aquilion 16-slice CT scanner. The specimen was scanned at a slice thickness of 0.500 mm, 120 kV, and 350 Ma. Raw scan data were exported in DICOM format and then imported into Mimics 17.0. The data comprise 1,521 slices each with dimensions of 512 × 512 pixels and a pixel spacing of 0.969 mm. The resolution of the CT scan data was high enough to record much of the internal structure of the maxilla.

The electronic version of this article in Portable Document Format (PDF) will represent a published work according to the International Commission on Zoological Nomenclature (ICZN), and hence the new names contained in the electronic version are effectively published under that Code from the electronic edition alone. This published work and the nomenclatural acts it contains have been registered in ZooBank, the online registration system for the ICZN. The ZooBank Life Science Identifiers (LSIDs) can be resolved and the associated information viewed through any standard web browser by appending the LSID to the prefix http://zoobank.org/. The LSID for this publication is: urn:lsid:zoobank.org:pub:7022E830-4C36-470A-BF78-10BE500E1519. The online version of this work is archived and available from the following digital repositories: PeerJ, PubMed Central and CLOCKSS.

## Systematic Paleontology

ARCHOSAURIA Cope, 1870 *sensu* Gauthier & Padian, 1985
SUCHIA Krebs, 1974 *sensu* Sereno, 1991
RAUISUCHIDAE von Huene, 1942 *sensu* Nesbitt, 2011
*Vivaron haydeni* gen. et sp. nov.

**Derivation of name:** *Vivaron*, named for the mythical 30-foot rattlesnake demon believed to haunt Orphan Mesa at Ghost Ranch (Poling-Kempes, 2005); *haydeni*, in honor of John Hayden, who discovered the Hayden Quarry from which the type and referred material was collected.

**Holotype:** right maxilla (GR 263).

**Referred material:** left premaxilla (GR 391), left maxilla (GR 186), left jugal (GR 641), right quadrate (GR 639), right ectopterygoid (GR 640), right ectopterygoid (GR 451). We tentatively refer to this taxon a right ilium (GR 638), right ilium (GR 642), tooth (GR 560), tooth (GR 664).

**Type Horizon:** Petrified Forest Member, Chinle Formation (Late Triassic: middle Norian, ∼212 Ma) (Irmis et al., 2011).

**Figure 1:**
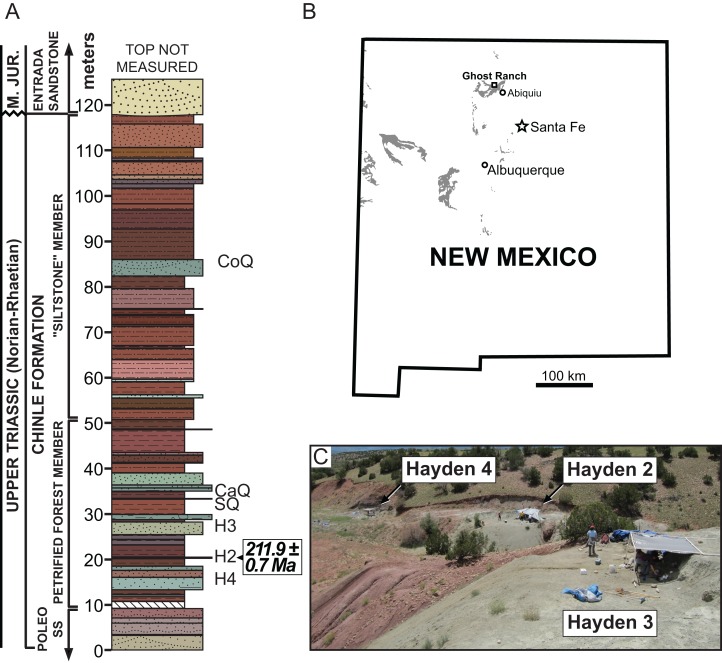
Stratigraphic and geographic location of the Hayden Quarry. (A) Stratigraphic section showing the location of major Ghost Ranch vertebrate fossil sites (adapted from Whiteside et al., 2015), (B) Map of New Mexico with Triassic exposures in grey (adapted from Irmis et al., 2011), and (C) Locality photo of the Hayden Quarry showing the relative locations of paleochannels 2, 3, and 4. Abbreviations: CaQ, Canjilon Quarry; CoQ, Coelophysis Quarry; H2, Hayden Quarry 2; H3, Hayden Quarry 3; H4, Hayden Quarry 4; SQ, Snyder Quarry.

**Type Locality:** Hayden Quarry 2 paleochannel, Ghost Ranch, Rio Arriba County, New Mexico, USA. Referred material is from Hayden Quarry paleochannels 2, 3, and 4; all three paleochannels are geographically within 30 m of each other and stratigraphically within 15 m of each other (Fig. 1).

**Diagnosis:** *Vivaron haydeni* differs from all other members of Rauisuchidae (*sensu* Nesbitt, 2011) by the presence of two prongs at the posterior extent of the maxilla for articulation with the jugal. *V. haydeni* and *Teratosaurus suevicus* (NHMUK 38646) share the following unique combination of character states: the antorbital fossa is bordered ventrally by a distinct margin that is the lateral surface of the maxilla rather than a raised ridge as in *Postosuchus kirkpatricki* (TTU-P 9000) and *Polonosuchus silesiacus* (ZPAL AbIII/563), a ventral margin that is straight rather than sinuous as in *Pos. kirkpatricki* (TTU-P 9000) and *Pol. silesiacus* (ZPAL AbIII/563), and a well-defined dental groove. In comparison with *Pol. silesiacus* (ZPAL AbIII/563), the maxilla of *V. haydeni* has a straighter anterior border, an antorbital fossa that extends further ventrally onto the ascending process, completely fused interdental plates, and a palatal process that does not extend as far medially. *Rauisuchus tiradentes* (BSPG AS XXV 60) does not preserve a maxilla but preserves four premaxillary alveoli, as do the premaxillae of *Saurosuchus galilei*, *Fasolasuchus tenax*, *Pol. silesiacus*, and *Pos. kirkpatricki*, whereas the referred premaxilla of *V. haydeni* (GR 391 contains five alveoli, the same state as in many early crocodylomorphs (Nesbitt, 2011).

**Remarks:** Our description is based on 11 skeletal elements discovered in the Hayden Quarry; this site comprises three separate but closely associated paleochannels at Ghost Ranch in northern New Mexico, USA (Fig. 1) (see Irmis et al., 2007; Irmis et al., 2011; Nesbitt et al., 2009a; Nesbitt et al., 2009b; Whiteside et al., 2015; Pritchard et al., 2015 for more information about this locality and its geology). Material from Hayden paleochannel 2 includes: the holotype maxilla (GR 263), GR 186, GR 391, GR 639, and GR 640. GR 263 is very thin, having been flattened mediolaterally during preservation but is the same length anteroposteriorly as the referred left maxilla (GR 186). The right quadrate (GR 640) is complete but has been crosscut by a small, mineralized fault plane, resulting in its collection as two separate pieces that do not fit back together precisely. All of the skull elements found in Hayden paleochannel 2 are about the same size, lack overlapping elements, and were found within a few meters of each other. Therefore, we hypothesize that they belong to the same individual, yet we only designate the nearly complete right maxilla as holotype. Two right ilia (GR 638 and GR 642) were found in Hayden paleochannel 3 and are assignable to Rauisuchidae using apomorphies, so we tentatively refer them to *Vivaron*. Hayden paleochannel 4 yielded additional disarticulated cranial material assignable to Rauisuchidae, so these specimens are is also tentatively assigned to the new taxon (GR 451, GR 560, GR 641, and GR 664).

## Comparative Morphological Description

### Maxilla (holotype, GR 263)

**Figure 2:**
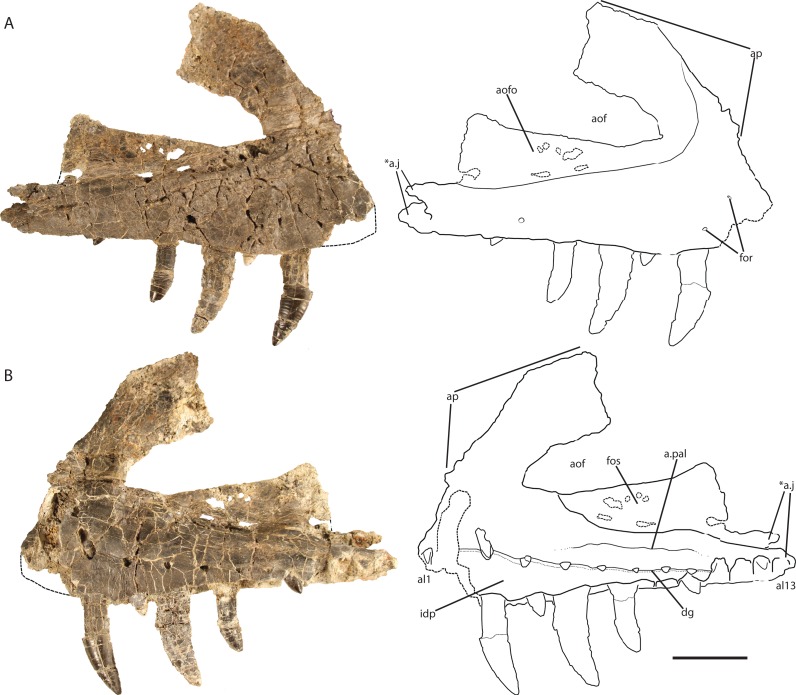
Holotype right maxilla of *Vivaron haydeni* gen. et. sp. nov. (GR 263) in (A) lateral and (B) medial views (with interpretive drawings). Abbreviations: a, articulation; al, alveolus; aof, antorbital fenestra; aofo, antorbital fossa; ap, ascending process; dg, dental groove; for, foramen; fos, fossa; idp, interdental plate; j, jugal; pal, palatine; *indicates autapomorphy. Scale bar = 5 cm.

The right maxilla (Fig. 2) is dorsoventrally tall and mediolaterally compressed with a sub-rectangular main body in lateral view. Although nearly complete, the anteriormost portion is not preserved, and therefore details of its articulation with the premaxilla and the presence of a subnarial fenestra are not clear. The dorsal and ventral margins are sub-parallel across the length of the maxilla from the posterior end to the base of the ascending (= dorsal) process at the anterior end. The ventral margin is nearly straight dorsal to alveoli seven through 13 and gently convex above alveoli three through six.

**Figure 3:**
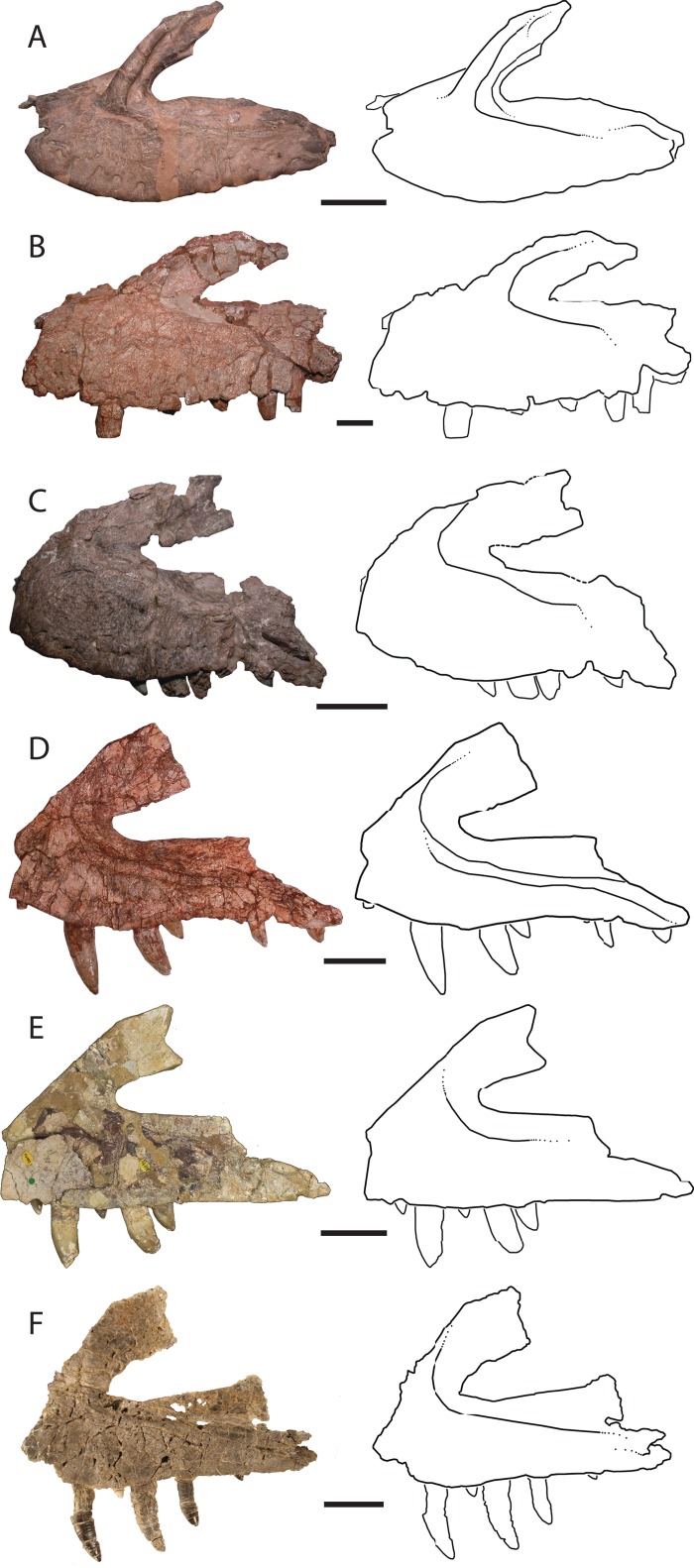
Left lateral views and interpretive drawings of the maxillae of (A) *Batrachotomus kupferzellensis* (SMNS 52970), (B) *Fasolasuchus tenax* (PVL 3851), (C) *Polonosuchus silesiacus* (ZPAL AbIII/563), (D) *Postosuchus kirkpatricki* (TTU-P 9000), (E) *Teratosaurus suevicus* (NHMUK 38646; reversed), and (F) *Vivaron haydeni* gen. et. sp. nov. (GR 263; reversed) emphasizing the antorbital fossa. Scale bars = 5 cm.

Ventral to the ascending process, the lateral surface of the maxilla possesses two nutrient foramina. The anterior foramen is just dorsal to the second alveolus and measures 2 mm in diameter, whereas the posterior foramen is 3.5 mm in diameter and is just dorsal to the third alveolus (Fig. 2A). The ascending process is mediolaterally thin, plate-like, and forms the dorsal and anterior borders of the antorbital fenestra. The process is thicker ventrally where it contacts the main body of the maxilla, and anteriorly, with edges that thin dorsally and posteriorly. The preserved portion of the anterodorsal edge is straight. The ascending process extends posterodorsally at about 40° to the ventral border of the antorbital fenestra and is anteroposteriorly wide across the entire length, a character state shared with *T. suevicus* (NHMUK 38646), *Pos. kirkpatricki* (TTU-P 9000), and *Pol. silesiacus* (ZPAL AbIII/563) but differing from *Batrachotomus kupferzellensis* (SMNS 52970), *Fasolasuchus tenax* (PVL 3851), and *Saurosuchus galilei* (PVSJ 32) (Fig. 3), and poposauroids (e.g., Nesbitt et al., 2013; Parker & Nesbitt, 2013). The ascending process widens dorsoventrally towards its posterior edge where it would contact the lacrimal, which slightly decreases the angle between the ascending process and ventral border of the antorbital fenestra.

The ascending process forms the rounded dorsal edge of the antorbital fenestra, and the dorsal border of the main body of the maxilla forms the straight ventral edge of the fenestra. The anterior half of the antorbital fenestra is wedge-shaped, widening posteriorly and tapering in dorsoventral depth anteriorly similar to the condition in *S. galilei*, *B. kupferzellensis*, *F. tenax*, *T. suevicus*, *Pos. kirkpatricki*, and *Pol. silesiacus* (Nesbitt, 2011). Much of the lateral surface of the ascending process forms part of the antorbital fossa, which then extends posteriorly from the base of the ascending process to where the maxilla terminates at its contact with the lacrimal and jugal. The antorbital fossa widens dorsoventrally towards the jugal process of the maxilla as this process itself narrows dorsoventrally; as a result, the fossa occupies a larger proportion of the maxilla as it extends posteriorly along the element. The shape of the antorbital fossa is similar to that of *T. suevicus* (NHMUK 38646) and *Pos. kirkpatricki* (TTU-P 9000) in that the fossa extends dorsally onto the ascending process anteriorly, and also extends posteriorly along the entire length of maxilla (Fig. 3). This differs from the antorbital fossa of *Pol. silesiacus* (ZPAL AbIII/563), which does not extend as far dorsally onto the ascending process or as far posteriorly along the jugal process of the maxilla, and also has a sinuous ventral margin (Fig. 3). The antorbital fossa of other closely related loricatans (e.g., *Saurosuchus galilei* (PVSJ 32), *Batrachotomus kupferzellensis* (SMNS 52970), and *Fasolasuchus tenax* (PVL 3851)) extends dorsally onto the posterior portion of the ascending process only rather than onto the entire ascending process as in rauisuchids (Fig. 3).

The posterior portion of the maxilla is laterally expanded with two prongs that comprise the articulation with the jugal (Fig. 2). The ventromedially-positioned prong also houses the posteriormost four alveoli, and is both mediolaterally thicker and dorsoventrally taller than the lateral prong. The dorsolateral prong is a thin, wing-like projection that originates posteroventral to the antorbital fenestra and extends posteromedially. The two prongs are slightly separated, creating a slot for articulation with the jugal. This morphology is autapomorphic for *Vivaron haydeni*, whereas other rauisuchids (*Pos. kirkpatricki* (TTU-P 9000)*, Pol. silesiacus* (ZPAL AbIII/563), and *T. suevicus* (NHMUK 38646)) have only a single prong (Fig. 3), which is homologous to the ventromedial prong in *V. haydeni*.

The palatal process on the anterior portion of the medial surface of the maxilla of *V. haydeni* is not preserved, and the bone and interdental plates covering the first and second alveoli are missing as well. However, it is clear that the maxilla does widen medially over the second alveolus, indicating that the palatal process was present. The medial surface of the maxilla possesses a depression just ventral to the antorbital fenestra; this structure mirrors the shape of the antorbital fossa on the lateral side. This medial fossa extends posteriorly from dorsal to the fifth alveolus and is bordered ventrally by the rounded, raised portion of the maxilla until the tenth alveolus (Fig. 2B). Poor preservation of the thin portion of bone that forms the fossa makes it difficult to interpret whether an “infraorbital foramen” (*sensu* Galton, 1985) is present on the surface of the bone forming the fossa, as in *T. suevicus, B. kupferzellensis*, *Pos. kirkpatricki*, *Arganasuchus* (ALM 1), and *Arizonasaurus* (Brusatte et al., 2009). The medial fossa terminates posteriorly where the maxilla contacts the lacrimal and jugal. The posterior margin of the fossa is poorly preserved and its exact morphology is not clear. Just ventral to the fossa, an articular surface is preserved as a ridge and groove that parallels the ventral edge of the medial fossa as in *T. suevicus* (NHMUK 38646). The position and shape of this scar in *V. haydeni* corresponds to the articulation for the palatine in *Pos. kirkpatricki* (Weinbaum, 2011).

In medial view, a well-defined “dental groove” (*sensu* Galton, 1985) separates the dorsal part of the maxilla from the interdental plates in medial view (Fig. 2B). The groove connects foramina through which some replacement teeth are visible. This groove is dorsally convex between each foramen. The dental groove on the medial surface of *V. haydeni* connects each alveolus, and marks a distinctive step between the medial surface of the maxillary body and the interdental plates, a condition also present in, and previously considered autapomorphic for, *T. suevicus* (NHMUK 38646) (Brusatte et al., 2009). However, the dental groove in GR 263 is more sinuous than that of *T. suevicus* (NHMUK 38646). Centered dorsal to each alveolus in *V. haydeni* are seven preserved triangular foramina from alveolus three through nine. Posteriorly, the distance between the foramina and the ventral edge of the maxilla decrease in distance. All of the interdental plates are fused in *V. haydeni*, *Pos. kirkpatricki*, *T. suevicus*, and *F. tenax*, though only the posterior half of the interdental plates are fused in *Pol. silesiacus* (Nesbitt, 2011). There are minute nutrient foramina on the medial surfaces of the interdental plates, and the interdental plates are nearly square and decrease in dorsoventral height posteriorly from alveolus three onwards. Poor preservation has destroyed the plates dorsal to alveolus one, two, and ten to thirteen.

**Figure 4:**
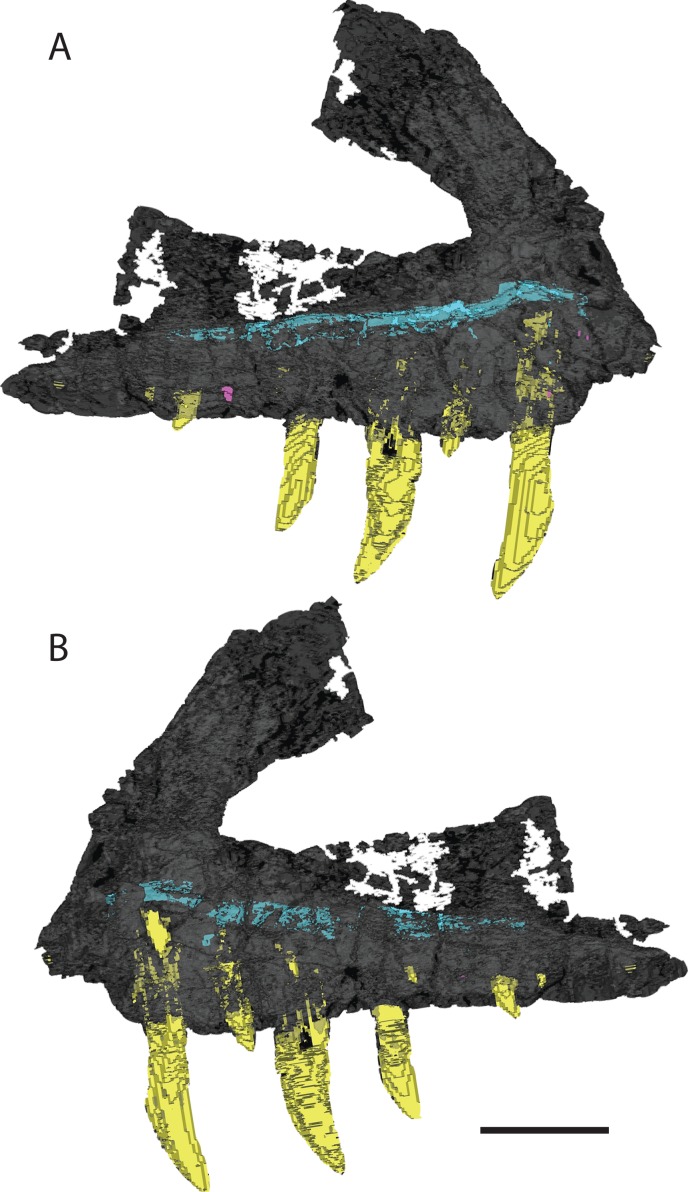
3D visualization of CT scan data of holotype right maxilla of *Vivaron haydeni* gen. et. sp. nov. (GR 263) in (A) lateral and (B) medial views with bone depicted in gray, teeth in yellow, and trigeminal nerve pathway in blue. Scale bar = 5 cm.

There are 13 alveoli and 12 teeth preserved in the right maxilla. A count of 13 alveoli is similar to *Pos. kirkpatricki* (TTU-P 9000) and *T. suevicus* (NHMUK 38646) but not *Pol. silesiacus* (ZPAL AbIII/563), which has 11 preserved alveoli. The first and second alveoli of *V. haydeni* are damaged, with the medial wall missing from the ventral margin to the base of the ascending process. The first alveolus is smaller than the second, and the alveoli decrease in size posteriorly from the second alveolus. Five erupted teeth are visible in lateral view (Fig. 2A). The two largest teeth are in the third and fifth alveoli. Another large tooth was shifted post-mortem from either the sixth or the seventh alveolus and sits in between those two alveoli. Two smaller, erupted teeth are present in the fourth and ninth alveoli. The lack of preservation of interdental plates has revealed replacement teeth in the first, 10th, and 12th alveoli, whereas CT data reveal the presence of four more replacement teeth, dorsal to alveolus three, four, five, and seven (Fig. 4). The replacement teeth are developing medially and parallel to the erupted teeth. The morphology of the individual teeth is described below.

### Maxilla (GR 186)

**Figure 5:**
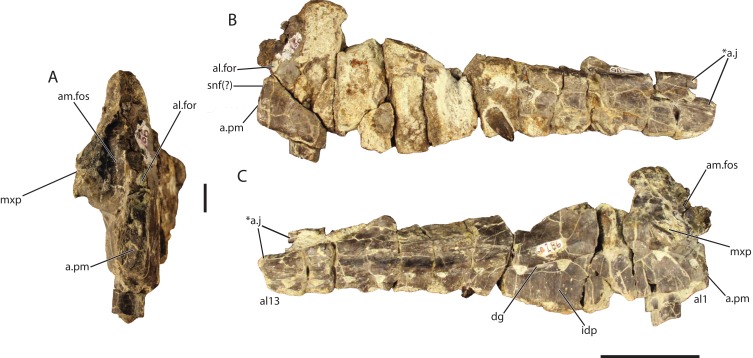
Referred left maxilla of *Vivaron haydeni* gen. et. sp. nov. (GR 186) in (A) anterior, scale bar = 1 cm (B) lateral, and (C) medial views. Abbreviations: a, articulation; al, alveolus; al.for, anterolateral foramen; am.fos, anteromedial fossa; ap, ascending process; dg, dental groove; for, foramen; idp, interdental plate; j, jugal; mxp, palatal process of the maxilla; pm, premaxilla; snf, subnarial fenestra; *indicates potential autapomorphy. Scale bar = 1 cm in (A) and 5 cm in (B) and (C).

The ascending process of the left maxilla (Fig. 5) has broken away, but the preserved portion is nearly identical to GR 263, and could belong to the same individual. The holotype and GR 186 share the presence of 13 maxillary alveoli, a well-defined dental groove, fused interdental plates, the lack of a laterally expanded lateral ridge, and the autapomorphy of two posteriorly directed prongs at the posterior end, indicating that they are referable to the same species.

Unlike the holotype, the anterior margin and palatal process of GR 186 are preserved. Ventral to the ascending process, the anterior margin of the maxilla is nearly vertical at its ventral termination, similar to *T. suevicus* (NHMUK 38646); this condition contrasts with the more posterodorsally angled anterior margin of the maxilla in *Pos. kirkpatricki* (TTU-P 9000) and the convexly rounded anterior margin in *Pol. silesiacus* (ZPAL AbIII/563). On the medial surface, the palatal process is broken at its anterior edge, but the preserved portion extends anteroventrally from its origination dorsal to the third alveolus. The medial surface of the process displays a groove that extends anteroventrally from the posterior portion of the palatal process towards the anterior portion of the main body of the maxilla. The placement and orientation of the palatal process are similar to those of *T. suevicus* (NHMUK 38646) and *Pos. kirkpatricki* (TTU-P 9000). In both GR 186 and *T. suevicus* (NHMUK 38646), ventral to the palatal process, the dental groove deflects anteroventrally between the first and second alveoli (Brusatte et al., 2009).

The palatal process overhangs the medial surface of the maxilla, forming an anteromedial foramen (Figs. 5A and 5C). This foramen, described as the ‘rostromedial foramen’ in *T. suevicus* by Brusatte et al. (2009), is also present in *Pos. kirkpatricki* (TTU-P 9000) and *Pol. silesiacus* (ZPAL AbIII/563) as well as the large non-rauisuchid paracrocodylomorphs *Fasolasuchus* (PVL 3851) and *Batrachotomus* (Gower, 1999). The anteromedial foramen does not extend posteriorly into the maxilla in GR 186 and is a fossa rather than the foramen described by Brusatte et al. (2009). The anterior surface of the maxilla also preserves an anterolateral foramen (Figs. 5A and 5B), (described as the ‘rostrolateral foramen’ in *T. suevicus* by Brusatte et al., 2009), which is also present in *Pos. kirkpatricki* (TTU-P 9000) and *Pol. silesiacus* (ZPAL AbIII/563) as well. These foramina may be present in *Batrachotomus*, and it is difficult to determine their presence in *Saurosuchus*, *Fasolasuchus*, and other loricatans because the feature is not described or figured in the literature.

GR 186 possesses 13 alveoli. The anteriormost alveolus is notably smaller than the following alveoli, a character state shared with *T. suevicus* (NHMUK 38646) and *Pos. kirkpatricki*, though not with *Pol. silesiacus* (Weinbaum, 2011). *V. haydeni* differs from *T. suevicus* (NHMUK 38646) in that the second alveolus in GR 186 contains a much larger tooth.

### Premaxilla (GR 391)

**Figure 6:**
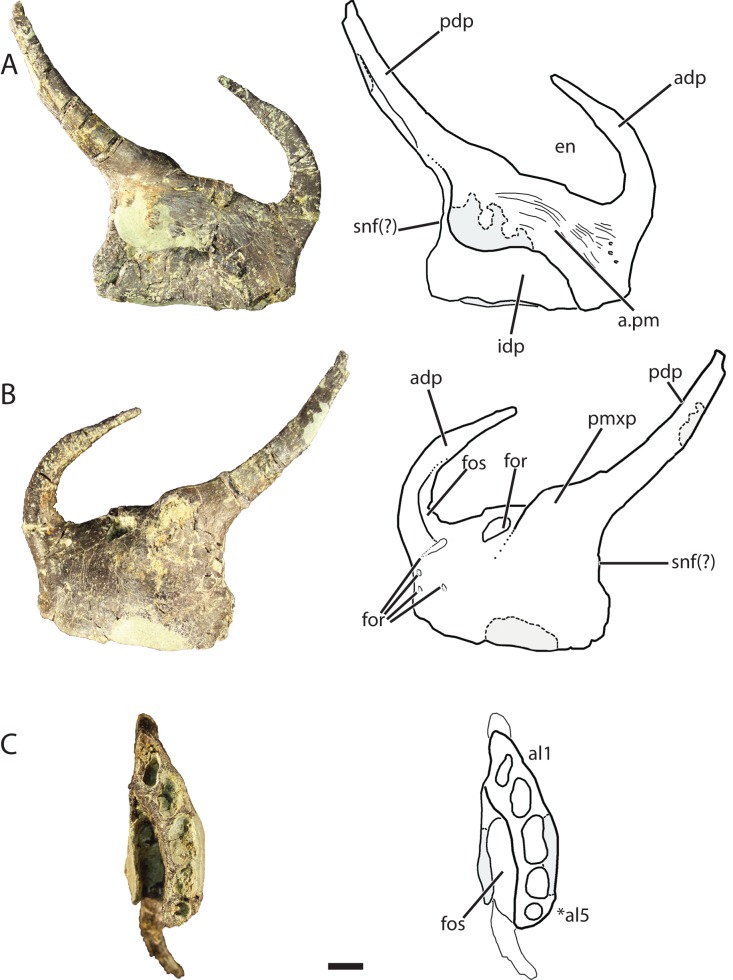
Referred left premaxilla of *Vivaron haydeni* gen. et. sp. nov. (GR 391) in (A) medial, (B) lateral, and (C) ventral views (with interpretive drawings). Abbreviations: a, articulation; adp, anterodorsal process; al, alveolus; en, external naris; for, foramen; fos, fossa; idp, interdental plate; pdp, posterodorsal process; pm, premaxilla; pmxp, premaxillary protuberance; snf, subnarial fenestra; *indicates potential autapomorphy. Scale bar = 1 cm.

The left premaxilla (Fig. 6) comprises a sub-rectangular main body with complete anterodorsal (= nasal) and posterodorsal (= maxillary) processes. The premaxilla is slightly longer anteroposteriorly than it is tall dorsoventrally and narrows anteriorly in the dorsoventral direction across its entire length, more so than the premaxilla of *Pos. kirkpatricki* (TTU-P 9000) and *Pol. silesiacus* (ZPAL AbIII/563). There are four small nutrient foramina on the anterolateral surface of the premaxilla. Two of those foramina are ventral to the anterodorsal process, whereas the other two are slightly posterior to these.

The anterodorsal process of the premaxilla is shorter than the anteroposterior length of the premaxilla, a character state described by Nesbitt (2011) as present in nearly all archosauriforms, but it is broken at the tip like the anterodorsal processes in *Pos. kirkpatricki* (TTU-P 9000) and *Pol. silesiacus* (ZPAL AbIII/563). The anterodorsal process in GR 391 rises from the premaxilla body and curves posteromedially to where it would contact the nasal as in the condition described for *Pos. kirkpatricki* (TTU-P 9000) (Weinbaum, 2011). The anterodorsal process forms the anterior border and anterodorsal corner of the external naris.

There are two prominences on the dorsolateral surface of the premaxilla (Fig. 6B) that represent a shared character state with *Pos. kirkpatricki* (Weinbaum, 2011) and *R. tiradentes* (BSPG AS XXV 60; previously described as autapomorphic for this taxon by Lautenschlager & Rauhut (2015)). In *R. tiradentes* these begin as knob-like thickenings at the base of the posterior process of the premaxilla and are notably more rugose than the premaxillary body (Lautenschlager & Rauhut, 2015). In contrast, the prominences on GR 391 are less well-defined than in *Pos. kirkpatricki* (TTU-P 9000) and *R. tiradentes* (BSPG AS XXV 60) and have no groove dividing them. The first prominence in GR 391 extends from the middle of the dorsal margin of the premaxilla to the base of the posterodorsal process. It is marked by the presence of a large foramen (Fig. 6B) (identified as a resorption pit by Weinbaum, 2011) that has been widened by preservational damage. The foramen in GR 391 opens medially, extending deep into the body of the premaxilla. The posterodorsal process projects posterodorsally from the second prominence, likely separates the maxilla and external naris, and forms the posterior and posteroventral borders of the ovate and anteroventrally angled external naris. This external naris shape and angle were described as subterminal by Weinbaum (2011), and are character states shared with *Pos. kirkpatricki* (TTU-P 9000) and *R. tiradentes* (BSPG AS XXV 60); it is difficult to determine if this is also shared with *Pol. silesiacus* because of incomplete preservation. The posterior surface of the posterodorsal process of GR 391 is concave. Ventral to the posterodorsal process, the posterior surface of the premaxilla is indented, indicating the possibility of a small subnarial foramen, a character state present in *Pol. silesiacus, Pos. kirkpatricki,* and *R. tiradentes* (Nesbitt, 2011). The anteroventral margin of the external naris of GR 391 is bordered by a shallow fossa that spans from the anterodorsal process to the base of the posterodorsal process (Fig. 6B). This depression is also present in *Pol. silesiacus* (ZPAL AbIII/563), *B. kupferzellensis* (SMNS 80260), and *Pos. kirkpatricki* (TTU-P 9000).

The medial surface of the premaxilla preserves the premaxillary symphysis and a deep fossa located posterolateral to the symphysis and ventral to the base of the posterodorsal process (Fig. 6C). This fossa is also present in *R. tiradentes* (BSPG AS XXV 60) and *Pos. kirkpatricki* (TTU-P 9000). The premaxillary symphysis forms an anterodorsally-oriented shelf that overhangs the fossa on the medial surface of the premaxilla from the second to the fifth alveolus (Fig. 6A). The symphysis is flat and covered with small foramina and grooves that cover the anterior and ventral portions of the premaxilla from the base of the posterodorsal process to the anterodorsal process. The premaxillary interdental plates are fused.

There are five alveoli preserved in the premaxilla. The presence of five premaxillary alveoli in *V. haydeni* differs from the four alveoli present in all other rauisuchids (*Pos. kirkpatricki*, *Pol. silesiacus*, *R. tiradentes*) and their close relatives (*Batrachotomus*, *Fasolasuchus*, and *Saurosuchus*). A skull reconstruction of *Pos. kirkpatricki* (UCMP A269) figured in Long & Murry (1995: Fig. 121) shows a left premaxilla possibly preserving a fifth alveolus. In contrast, five or more premaxillary alveoli are present in early crocodylomorphs (e.g., *Redondavenator quayensis* (NMMNH P-25615) and *Hesperosuchus agilis* (CM 29894)) (Nesbitt, 2011). The anteriormost alveolus in GR 391 is oval and angled anterolaterally. The alveoli cross-sections become more sub-circular posteriorly. The third alveolus is the largest (13 mm across its longest axis and 6 mm across its shortest axis) and the fifth alveolus is the smallest (diameter of 4 mm).

### Jugal (GR 641)

**Figure 7:**
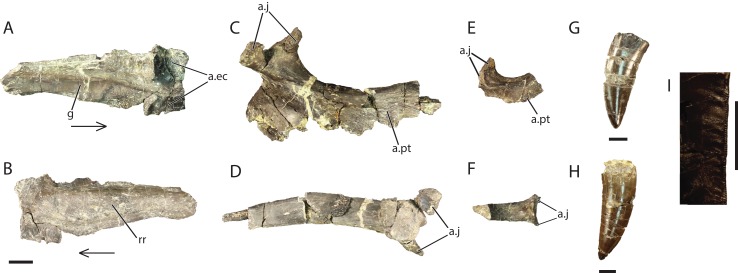
Referred cranial elements of *Vivaron haydeni* gen. et. sp. nov. Left jugal (GR 641) in (A) medial and (B) lateral views; right ectopterygoid (GR 640) in (C) dorsal and (D) lateral views; right ectopterygoid (GR 451) in (E) dorsal and (F) lateral views; tooth (GR 560) (G); tooth (GR 664) (H) and wrinkled enamel (I). Abbreviations: a, articulation; ec, ectopterygoid; g, groove; j, jugal; pt, pterygoid; rr, rugose ridge. Scale bars = 1 cm; arrows point anteriorly.

Only a small portion of the left jugal (Figs. 7A and 7B) is preserved, including the articular region for the ectopterygoid and the area posterior to it. The element is missing both the anterior and posterior processes but preserves a bulbous longitudinal ridge on the lateral surface. Among Archosauria, a jugal longitudinal ridge that is restricted to a bulbous ridge is only otherwise found in *Pos. kirkpatricki* (TTU-P 9000), *R. tiradentes* (BSPG AS XXV 63), and *Pol. silesiacus* (ZPAL AbIII/563) (Nesbitt, 2011). The ridge on GR 641 tapers posteriorly and has many small foramina on its surface. The medial surface is smooth. Anteriorly, there are two sockets for articulation with the double-headed ectopterygoid, a condition also present in the rauisuchids *Pos. kirkpatricki* (TTU-P 9000) and *R. tiradentes* (BSPG AS XXV 63), and in the crocodylomorph *Sphenosuchus acutus* (Walker, 1990). The dorsal articular surface for the ectopterygoid is separated posteriorly from the rest of the jugal by a medially directed process. The ventral articular surface is slightly angled in the ventrolateral direction. The medial surface of the jugal also has a groove that extends longitudinally along its length, arcing dorsally and separating the ectopterygoid articulations. This is also present in *Pos. kirkpatricki* (TTU-P 9000) and *R. tiradentes* (BSPG AS XXV 63).

### Quadrate (GR 639)

**Figure 8:**
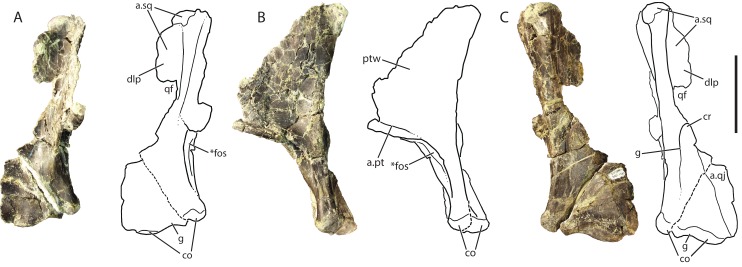
Referred right quadrate of *Vivaron haydeni* gen. et. sp. nov. (GR 639) in (A) anterior, (B) medial, and (C) posterior views (with interpretive drawings). Abbreviations: a, articulation; co, condyle; cr, crest; dlp, dorsolateral process; fos, fossa; g, groove; pt, pterygoid; ptw, pterygoid wing; qf, quadrate foramen; qj, quadratojugal; sq, squamosal; *indicates potential autapomorphy. Scale bar = 5 cm.

The right quadrate (Fig. 8) comprises a dorsoventral main shaft that widens ventrally into a triangle of bone in posterior view. This shaft is a ridge that terminates ventrally at the medial condyle of the glenoid. The anterior surface of the shaft has a concave surface that extends ventrally from the dorsal head and laterally onto the ventral body of the quadrate. The dorsalmost surface of the quadrate is rounded into a head that is the articular surface with the squamosal. The general shape of the quadrate, including the condyles and dorsoventrally oriented crest, is very similar to those of *Pos. kirkpatricki* (TTU-P 9000) and *Pol. silesiacus* (ZPAL AbIII/563).

The dorsal head of the quadrate possesses a posteriorly-oriented hook that is identical to that of *Pos. kirkpatricki* (TTU-P 9000) and may be present in *Pol. silesiacus* (ZPAL AbIII/563), though it is difficult to determine its presence because the feature is not described in the literature. Both a dorsolateral process and a pterygoid wing extend from the dorsal head in GR 639. The dorsolateral process extends from just ventral to the dorsal head to just dorsal to the quadrate foramen (discussed below). The dorsolateral process would articulate with the descending ramus of the squamosal, similar to the condition in *Pos. kirkpatricki* (TTU-P 9000). On the medial surface of GR 639, a large pterygoid wing projects anteromedially from the dorsal head to just dorsal to the anteromedially-facing fossa (described below). There is a horizontally oriented shelf on the ventral surface of the pterygoid wing, and just dorsal to the shelf is the articular surface for the pterygoid, as seen in *Pos. kirkpatricki* (TTU-P 9000).

The dorsolateral portion of the quadrate has a shallow and wide groove that extends laterally onto the dorsolateral wing. Just ventral to the dorsolateral wing, the medial portion of the quadrate foramen is present. There is a dorsoventrally oriented crest just ventral to the quadrate foramen and just dorsal to the articulation with the quadratojugal; this morphology results in a medially arcing concave surface (Fig. 8C). This crest is also present in *Pos. kirkpatricki* and *Pol. silesiacus* (Nesbitt, 2011). The concave surface is part of a groove that extends from the quadrate foramen to the ventral body of the quadrate above the medial condyle. In *Pos. kirkpatricki* (TTU-P 9000), the groove on the posterior surface of the distal end stretches to the medial surface of the quadrate, whereas in GR 639, the groove trends similarly, arcing medially and ventrally, but does not extend to the medial surface of the quadrate. The distal articular surface for the glenoid comprises two condyles separated by a shallow groove that trends anteromedially (Figs. 8A and 8C). The articulation with the quadratojugal is a shelf on the lateral surface of the ventral portion of the quadrate that is separated from the rest of the quadrate by a small, sharp ridge.

The ventromedial surface of the quadrate has a deep fossa (Figs. 8A and 8B) just ventral to the shelf on the pterygoid ramus. The fossa opens anteromedially, is surrounded on both sides by ridges, and shallows ventrally to a groove that trends towards the medial condyle. This characteristic has not been commented upon previously but may be present in *Pos. kirkpatricki* and *B. kupferzellensis*, though it is not described or figured in the literature.

### Ectopterygoid (GR 640; GR 451)

The following descriptions refer to the right ectopterygoid, GR 640 (Figs. 7C and 7D), because the other right ectopterygoid GR 451 (Figs. 7E and 7F) is less complete and pertains to a smaller individual (only 2.5 cm long anteroposteriorly, compared to 9 cm long in GR 640). Besides their relative sizes, the only noticeable difference between the two specimens is that the lateral surface of GR 451 is concave in the center.

The ectopterygoid is ‘J’-shaped with a thickened anterior head and a tapering posterior process that arches dorsally and anteriorly. This is in contrast to the ectopterygoid of *Pos. kirkpatricki* (TTU-P 9000), which only arcs anteriorly. The head of the ectopterygoid of *V. haydeni* displays both dorsal and ventral processes (Figs. 7C–7F) that are likely articular surfaces for the jugal, similar to *Pos. kirkpatricki*, *Pol. silesiacus*, *Batrachotomus*, *Sphenosuchus*, and *Hespersuchus “agilis”* (Nesbitt, 2011). It is difficult to determine if there is a groove separating these two processes in GR 640 because poor preservation has eliminated much of the surface where the groove is expected. The dorsal process possesses a groove on its ventral surface that extends onto the dorsal surface of the ectopterygoid.

The ectopterygoid is concave ventrally and laterally. The anterior portion of the ectopterygoid extends medially as a thin flange that narrows dorsoventrally. Sutural surfaces, thin scars filled with small pitting, trend anteroposteriorly in the posterodorsal region of the ectopterygoid. The posteromedial surface of the ectopterygoid has a raised ridge anterior to the jugal contact. There is a large flange on its medial side that appears to contribute to a large portion of the pterygoid flange, a shared character state of Archosauriformes (Nesbitt, 2011). The posterior process of the ectopterygoid narrows medially. Some small scars are visible on the medial surface of the posterior process where the pterygoid would contact the ectopterygoid.

### Dentition

Both the isolated (GR 560, GR 664) (Figs. 7G and 7H) and in situ maxillary teeth (Fig. 2) are recurved at the tip, oval in cross-section, mediolaterally compressed, have lineations trending dorsoventrally, and are serrated on both their anterior and posterior carinae. Serration density averages three serrations per millimeter, similar to *Pos. kirkpatricki* (Weinbaum, 2011); this is less dense than the 4–5 serrations per millimeter reported by Lautenschlager & Rauhut (2015) for *R. tiradentes*. The isolated teeth are similar in size to the two largest maxillary teeth of GR 263. The largest in situ maxillary tooth from GR 263, and both isolated teeth (GR 560 and GR 664) have wrinkled enamel along the posterior carina (Fig. 7I). This character state is also present in *Batrachotomus* (SMNS 52970), other rauisuchids, and theropod dinosaurs (Brusatte et al., 2009). The wrinkles extend anteriorly over the posterior half of the tooth and dorsoventrally along the entire carina. Though the isolated teeth are consistent with the maxillary teeth of *V. haydeni*, we acknowledge that serrated, mediolaterally compressed, recurved teeth are plesiomorphic for Archosauria.

### Ilium (GR 638; GR 642)

**Figure 9:**
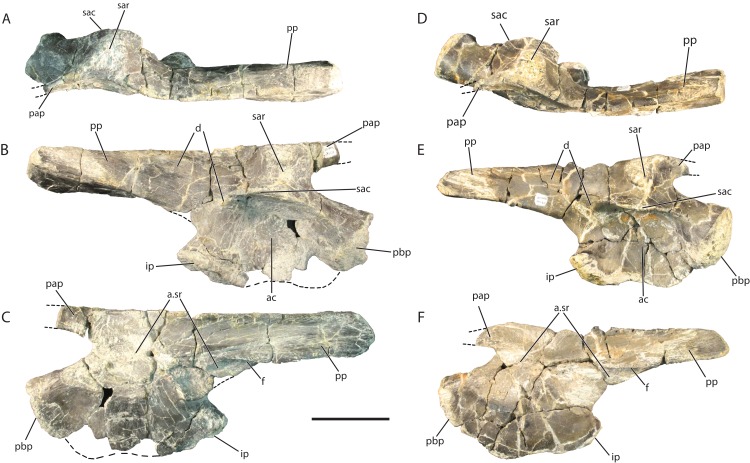
Referred right ilia of *Vivaron haydeni* gen. et. sp. nov. GR 638 in (A) dorsal, (B) lateral, and (C) medial views; GR 642 in (D) dorsal, (E) lateral, and (F) medial views. Abbreviations: a, articulation; ac, acetabulum; d, depression; f, flange; ip, ischial peduncle of the ilium; pap, preacetabular process; pbp, pubic peduncle of the ilium; pp, postacetabular process; sac, supra-acetabular crest; sar, supra-acetabular ridge; sr, sacral. Scale bar = 5 cm.

The larger right ilium, GR 638, is 22 cm in total anteroposterior length (measured from the posterior point on the postacetabular process to the most anterior point on the pubic peduncle), whereas the smaller right ilium, GR 642, is 18 cm long (Fig. 9). The ilia preserve an acetabulum on the lateral surface, with ventral articulations for the ischium and pubis, as well as dorsal preacetabular and postacetabular processes. The preacetabular process on both GR 638 and GR 642 is broken anteriorly, so it is impossible to determine whether or not it extends anterior to the acetabulum, as in *Pos. kirkpatricki* and crocodylomorphs (Nesbitt, 2011). The preserved portion of the preacetabular process curves medially and narrows anteriorly. The preacetabular process is separated from the postacetabular process by a thick, vertical, laterally expanded ridge (Figs. 9A, 9B, 9D and 9E) dorsal to the supra-acetabular crest. This ridge is also present in SMNS 52972 (an ilium previously assigned to *Teratosaurus*; discussed below) and *Pos. kirkpatricki* (TTU-P 9002), and is present but less expanded in *S. galilei*, *B. kupferzellensis*, and members of Poposauroidea (Gower & Schoch, 2009; Nesbitt, 2011). The ilium is 3 cm thick mediolaterally at the supra-acetabular ridge dorsal to the acetabular crest in GR 638 and 2.5 cm mediolaterally in GR 642.

Laterally, the acetabulum is a deep, round depression that measures 5.5 cm high dorsoventrally in GR 638 and 5 cm high in GR 642. The dorsal edge of the acetabulum is formed by a laterally projecting supra-acetabular crest that overhangs the rest of the acetabulum (it is angled slightly ventrally GR 642 only). This rim defines the mediolateral width of the ilium; it extends anteriorly onto the lateral surface of the pubic peduncle, and has a small depression (Figs. 9B and 9D) at its posterior terminus. The ventral border of the ilium is convex along the ventral margin of the pubic peduncle and slightly concave along the same margin of the ischial peduncle. Overall, the ventral margin is mediolaterally thin and sinuous in lateral view in both GR 638 and GR 641, a feature shared with SMNS 52972, whereas the same region converges to a convex point in *Pos. kirkpatricki* (TTU-P 9002).

The postacetabular process of the ilium of *V. haydeni* comprises half the total anteroposterior length of the ilium, and tapers posteriorly. There are many small grooves trending longitudinally along the lateral surface of the postacetabular process which could be the muscle attachment site for the flexor tibialis externus (Schachner, Manning & Dodson, 2011). There is a small ridge on the postacetabular process dorsal to the ischial peduncle that is the dorsal border of a slight oval depression on the postacetabular process (Figs. 9B and 9D). The postacetabular process meets the acetabular region of the ilium at a more dorsal point than in the *Pos. kirkpatricki* (UMMP 7333) and SMNS 52972, with a clear separation of the ischial peduncle and the anteroventral-most part of the postacetabular process. The medial ridge of the postacetabular process has a medioventrally extending blade-like flange trending anteroposteriorly along its surface (Figs. 9C and 9F). In both GR 642 and SMNS 52972, the postacetabular process arcs medially at its base whereas in *Pos. kirkpatricki* (UMMP 7266) the postacetabular process is straighter. In lateral view, the dorsal edge of the postacetabular process of *V. haydeni* is flat, similar to SMNS 52972 but differing from *Pos. kirkpatricki* (UMMP-7333), in which the process expands slightly dorsally at its middle portion. In dorsal view, the dorsal border of the ilium is sinuous, similar to that of SMNS 52972 and *Pos. kirkpatricki* (TTU-P 9002).

The medial surface of the ilium of *V. haydeni* is smooth with the exception of the articular surfaces for sacral ribs. GR 638, GR 642, and *Pos. kirkpatricki* (TTU-P 9002) possess two sacral rib articular facets whereas SMNS 52972 appears to have two, but has been reported to have three (Galton, 1985). Of the two observed in *V. haydeni*, the first facet is medial to the thickened supra-acetabular ridge dorsal to the acetabular crest. The second sacral rib facet is located where the postacetabular process meets the acetabulum and extends onto the postacetabular process, ventral to the flange.

The referred ilia of *Vivaron haydeni* are very similar to SMNS 52972, the ilium previously referred to *T. suevicus* (Galton, 1985; Brusatte et al., 2009) in the shared presence of the following character states: presence of a distinct supra-acetabular ridge dorsal to the acetabular crest, two sacral rib articulations, a similar ventral acetabular edge, a postacetabular process arcing medially at its base, and a flat edge to the dorsal edge of the postacetabular process when in lateral view. The locality data for SMNS 52972, originally considered to be from the Middle Stubensandstein of Germany, cannot be confirmed so Brusatte et al. (2009) stated that it can only be considered as “Rauisuchia indet.” The inferred close relationship between *V. haydeni* and *T. suevicus* based on maxillary characters and the shared ilium features are consistent with assignment of the ilium SMNS 52972 to *T. suevicus*. However, such a referral must remain tentative given that the type material of both taxa (i.e., *T. suevicus* and *V. haydeni*) was not found associated with their respective putative ilia.

## Phylogenetic Analysis

### Methods

We used a modified version of the data set of Nesbitt (2011), consisting of 412 characters and 80 terminal taxa, to examine the phylogenetic relationships of *Vivaron* within Pseudosuchia. *Vivaron haydeni* was scored for 61 characters using the holotype and referred specimens. We also included *Teratosaurus suevicus,* which could only be scored for 23 characters (all maxillary). *Tikisuchus romeri*, though most likely a member of Rauisuchidae, was not included in the analysis because the material is incompletely described in the literature and none of the authors have observed the material first hand. A new state was added to character 26 (maxilla, lateral surface: (0) smooth; (1) sharp longitudinal ridge present; (2) bulbous longitudinal ridge present; (3) distinct dropoff to antorbital fossa (new)); this new character state is only scored as present in *V. haydeni* and *T. suevicus*. The scorings for the ilium of *Rauisuchus tiradentes* were changed to uncertainty (?) following Lautenschlager & Rauhut’s (2015) observation that the only known ‘*Rauisuchus*’ ilium (BSPG AS XXV 88) could not be confidently assigned to the species. The distributions of five additional characters within Pseudosuchia are not well-characterized with the current taxon sampling regime so they were not included in the analysis. These include: the presence or absence of lateral protuberances at the base of the posterior (= maxillary) premaxillary process of the premaxilla (as a possible character state in *Fasolasuchus tenax*, Rauisuchidae, and Crocodylomorpha); the presence or absence of a shallow fossa on the premaxilla bordering the anteroventral margin of the external naris (as a possible character state in *V. haydeni*, *Pos. kirkpatricki*, *Pol. silesiacus*, and *B. kupferzellensis*); the presence or absence of an anterolateral foramen on the anterior surface of the maxilla (as a possible character state in *V. haydeni*, *T. suevicus*, *Pos. kirkpatricki*, and *Pol. silesiacus*); the presence or absence of an anteromedially opening fossa on the ventromedial surface of the quadrate (as a possible character state in *V. haydeni*, *Pos. kirkpatricki*, and *B. kupferzellensis*); and the presence or absence of a posteriorly-oriented hook on the dorsal head of the quadrate (as a possible character state in *V. haydeni*, *Pos. kirkpatricki*, and *Pol. silesiacus*).

Five original terminal taxa were deleted (*Prestosuchus chiniquensis*, UFRGS 0156-T, UFRGS 152-T, *Lewisuchus admixtus*, and *Pseudogalosuchus major*) in the final analysis because they were combined into two separate terminal taxa, *Prestosuchus* (comprising *Prestosuchus chiniquensis*, UFRGS 0156-T, UFRGS 152-T) and *Lewisuchus/Pseudogalosuchus* within Avemetatarsalia. All characters were equally weighted and 19 were ordered (32, 52, 121, 137, 139, 156, 168, 188, 223, 243, 258, 269, 271, 291, 297, 328, 356, 371, 399). A maximum parsimony analysis was conducted using PAUP* version 4.0b10 (Swofford, 2002) using a heuristic tree search with 10,000 replicates (using random addition sequences) followed by tree bisection and reconnection (TBR) branch swapping. The analysis was run with the option ‘collapse branches if minimum length is zero.’ Character transformations were examined using ACCTRAN and DELTRAN optimizations to determine unambiguous synapomorphies as well as other possible synapomorphies.

### Results

**Figure 10:**
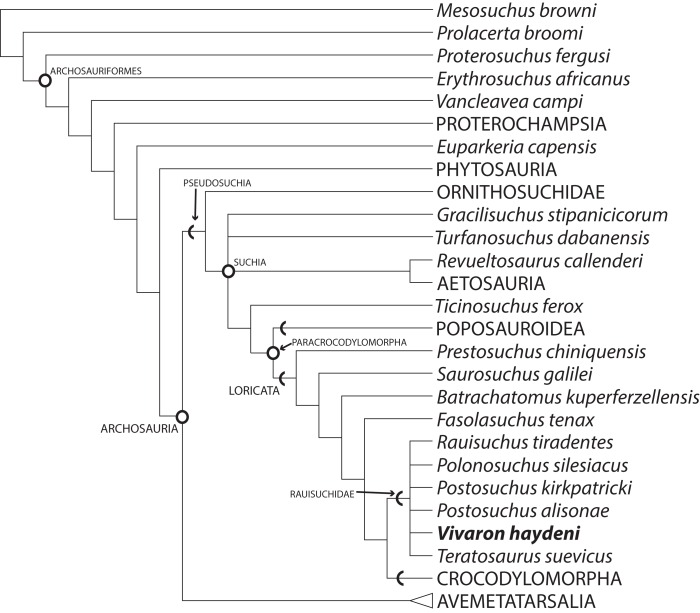
Strict consensus of Archosauria (80 taxa, 412 characters) highlighting relationships of *Vivaron haydeni* gen. et. sp. nov. within Rauisuchidae. Consensus of 3,240 MPTs of length 1,287. Circles = nodes; chevrons = stem groups.

Our analysis recovered 3,240 most parsimonious trees (TL = 1,287; CI = 0.3741; RI = 0.7751; RC = 0.2900) (Fig. 10) where *Vivaron haydeni* was recovered as a member of Rauisuchidae. Overall, the relationships of pseudosuchians are identical to those of a previous analysis (Nesbitt, 2011). The strict consensus recovered all members of Rauisuchidae in a polytomy (*R. tiradentes*, *Pol. silesiacus*, *Pos. kirkpatricki*, *Pos. alisonae*, *T. suevicus*, and *V. haydeni*); this clade was the sister taxon to Crocodylomorpha. Rauisuchidae is supported by the following unambiguous synapomorphies (those with an asterisk support placement of *V. haydeni* within Rauisuchidae; those with a dagger exhibit no homoplasy among the MPTs): a bulbous longitudinal ridge present on the lateral surface of the maxilla (character 26: state 2); a maxillary ascending process that remains wide across its length (29:1*); the dorsolateral margin of the anterior portion of the nasal having a distinct anteroposteriorly oriented ridge on the lateral edge (35:1); the anteroventral process of the squamosal present and contacting the postorbital bisecting the lower temporal fenestra (52:2); the presence of a longitudinal ridge on the lateral surface of the jugal that is rounded and bulbous (75:3*); the presence of a dorsoventrally oriented crest located on the posterior side of the quadrate (83:1*^†^); the large exit of cranial nerve VII (125:1^†^); palpebrals extensively sutured to each other and to the lateral margin of the frontals (149:1); and the ventral surface of the axis possessing two paramedian keels (180:1). Among those characters that can be scored for the taxon, *Vivaron haydeni* is differentiated from all other members of Rauisuchidae by the presence of five premaxillary teeth (6:2). The clade of *Postosuchus* + *Polonosuchus* in Nesbitt’s (2011) study was not recovered in our new analysis because the previous support for this group was based on character states (in the squamosal and cervical vertebrae) that could not be scored for *T. suevicus* and *V. haydeni*. A survey of the interrelationships within Rauisuchidae represented in the most parsimonious trees reveals nine highly variable arrangements because of missing data. The large missing data percentages of *V. haydeni* (85.2% missing) and *T. suevicus* (94.4%) cause the lack of resolution. Typically, we find *Pos. kirkpatricki* as sister taxon to *Pol. silesiacus* supported by a wide maxillary ascending process (26:2) and an asymmetrical distal articulation on metatarsal IV (391:1). We also find *R. tiradentes* as a sister taxon to all the other members of Rauisuchidae, supported by the absence of a deep pit on the posterodorsal corner of the lateral surface of the squamosal (57:0) and the absence of hypapophyses on the middle cervical vertebrae (192:0). Changing 13 characters of the ilium from scored states to uncertainty for *R. tiradentes* did not affect the outcome of the analysis in any considerable manner as *R. tiradentes* is still recovered as a member of Rauisuchidae. Removing *T. suevicus* from the analysis still resulted in a polytomy for Rauisuchidae, though with 1,080 MPTs (TL = 1,287; CI = 0.3753; RI = 0.7759; RC = 0.2912). An analysis with *V. haydeni* scored only from the holotype maxilla still recovered this taxon within a monophyletic, yet completely unresolved Rauisuchidae (3,240 MPTs; TL = 1,286; CI = 0.3753; RI = 0.7758; RC = 0.2911).

## Discussion

*Vivaron haydeni* is the second rauisuchid taxon discovered from the Triassic of the southwestern United States. Previously, despite spanning over a thousand kilometers of geographic distance and over 10 million years of time, nearly all southwestern United States rauisuchid crania and postcrania were assigned to a single species, *Postosuchus kirkpatricki* (e.g., Long & Murry, 1995; Zeigler, Heckert & Lucas, 2003). With the discovery of *V. haydeni*, we must be careful to make morphological comparisons with both *Pos. kirkpatricki* and *V. haydeni*, as well as other rauisuchid taxa, when determining the assignment of rauisuchid material from the southwestern United States. Assignment must be based primarily on observable apomorphies and not geographic distribution (Nesbitt & Stocker, 2008).

**Figure 11:**
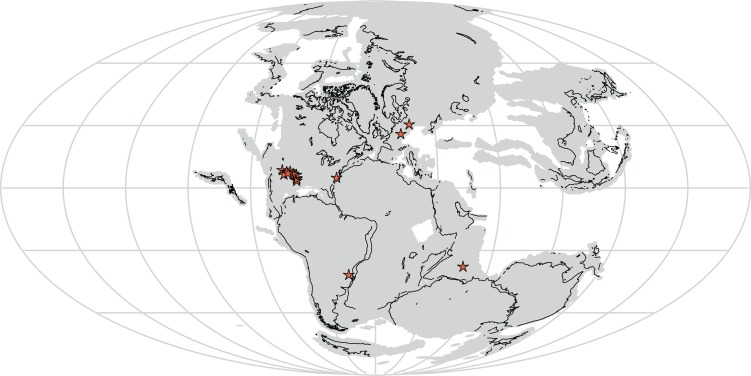
Distribution of Rauisuchidae across Pangea during the Late Triassic with each star marking a locality where rauisuchid material has been confirmed (25 stars present in the southwestern United States; generated from http://fossilworks.org/). The underlying source of the data is the Paleobiology Database (http://www.paleobiodb.org).

Furthermore, *Vivaron haydeni* increases known rauisuchid diversity worldwide, from five (*Pos. kirkpatricki*, *Pos. alisonae*, *R. tiradentes*, *Pol. silesiacus*, and *T. suevicus*) to six recognized species. Rauisuchids span paleolatitudes of approximately 5–40° north of and 3–60° south of the equator (paleolatitude estimates follow the apparent polar wander paths of Kent & Irving (2010) and Torsvik et al. (2012)) in what today is the southwestern and eastern United States, western Europe, India, and Brazil (Fig. 11) and are known from the late Carnian to mid-Norian (Benton, 1986; Nesbitt et al., 2013). Rauisuchids occurring in the late Carnian to early Norian include: *R. tiradentes* from Brazil, *Pos. alisonae* from the eastern United States, and *Pol. silesiacus* from Poland (Lautenschlager & Rauhut, 2015; Peyer et al., 2008; Sulej, 2005). *Tikisuchus romeri* from India is also late Carnian, and a potential additional member of Rauisuchidae (Chatterjee & Majumdar, 1987; Sulej, 2005; Nesbitt et al., 2013). Faunal associations are generally similar between the individual localities of *Pol. silesiacus*, *Pos. alisonae*, *R. tiradentes*, and *T. romeri* with the presence of some but not all of the following taxa: phytosaurs, aetosaurs, silesaurids, early dinosaurs, cynodonts, dicynodonts, and rhynchosaurs, though rauisuchids are the only taxa present in all four locations (Dzik & Sulej, 2007; Langer, 2005; Mukherjee & Ray, 2012; Olsen, Shubin & Anders, 1987). With the exception of *Pos. alisonae*, these taxa are all from 35° latitude or higher, in both the northern and southern hemispheres. In contrast, *Pos. alisonae* from the Deep River Basin of North Carolina, is from somewhere between 4°S and 0° paleolatitude depending on its exact age (Whiteside et al., 2011).

*Pos. kirkpatricki* is found in the Late Triassic from the early to mid-Norian in the southwestern United States at paleolatitudes of ∼5–10° N (Weinbaum, 2011). *T. suevicus*, previously the youngest taxon, is known from the mid-Norian of Germany at a paleolatitude of 35–40° N (Benton, 1986). *V. haydeni* is from a paleolatitude of ∼11° N and radioisotopically dated to the mid-Norian (∼212 Ma; see Irmis et al., 2011), making it possibly the youngest known rauisuchid. The temporal range of *Pos. kirkpatricki* and *V. haydeni* may differ from that described above if all rauisuchid material from southwestern United States is not assignable to *Pos. kirkpatricki*.

Though both are from the latter half of the Norian and morphologically similar, *T. suevicus* and *V. haydeni* are widely separated geographically and belong to very different faunal assemblages. In New Mexico, the tetrapod fauna of the Hayden Quarry includes metoposaurs, phytosaurs, aetosaurs, non-archosaur archosauromorphs, lagerpetids, silesaurids, and theropod dinosaurs (Irmis et al., 2007; Nesbitt et al., 2009b; Pritchard et al., 2015; Whiteside et al., 2015). At the mid-latitudes in Europe, *T. suevicus* occurs with abundant sauropodomorph dinosaurs in addition to turtles, phytosaurs, and aetosaurs (Irmis, 2011; Padian, 1988; Yates, 2003). Whereas the late Carnian to early Norian rauisuchid taxa shared similar faunal associations, *V. haydeni* and *T. suevicus* have somewhat dissimilar faunal associations. Thus, although the different members of Rauisuchidae were very similar morphologically, they were surrounded by and preyed upon different taxa in disparate environments over at least 16 million years.

## Supplemental Information

10.7717/peerj.2336/supp-1 Supplemental Information 1 Selected measurements for *Vivaron haydeni*. Selected measurements in cm unless otherwise specified.

10.7717/peerj.2336/supp-2 Supplemental Information 2 Phylogenetic matrix. Conducted using PAUP* version 4.0b10.

10.7717/peerj.2336/supp-3 Supplemental Information 3 Phylogenetic analysis results as .tre files. including the strict consensus, analysis without the ilia of *Rauisuchus*, analysis without *Teratosaurus*, and analysis without the referred material of *Vivaron*.

## Institutional abbreviations

BSPG: Bayerische Staatssammlung für Paläontologie und Geologie, Munich, Germany
CM: Carnegie Museum of Natural History, Pittsburgh, PA, USA
GR: Ghost Ranch Ruth Hall Museum of Paleontology, Abiquiu, New Mexico, USA
NHMUK: Natural History Museum, London, United Kingdom
NMMNH: New Mexico Museum of Natural History, Albuquerque, NM, USA
PVL: Instituto “Miguel Lillo,” Tucumán, Argentina
PVSJ: Division of Vertebrate Paleontology of the Museo de Ciencias Naturales de la Universidad Nacional de San Juan, Argentina
SMNS: Staatliches Museum für Naturkunde, Stuttgart, Germany
TTU-P: Texas Tech University Museum of Paleontology, Lubbock, TX, USA
UCMP: University of California Museum of Paleontology, Berkeley, California, USA
UMMP: University of Michigan Museum of Paleontology, Ann Arbor, MI, USA
ZPAL: Institute of Paleontology, Polish Academy of Sciences, Warsaw, Poland.

## Additional Information and Declarations

### Data Deposition

The following information was supplied regarding data availability: The raw data has been supplied as Supplementary Dataset Files.

### New Species Registration

The following information was supplied regarding the registration of a newly described species: *Vivaron haydeni* gen. et sp. nov. urn:lsid:zoobank.org:pub:7022E830-4C36-470A-BF78-10BE500E1519. Publication LSID: urn:lsid:zoobank.org:pub:7022E830-4C36-470A-BF78-10BE500E1519.
