# Supplementary material for: A new rauisuchid (Archosauria, Pseudosuchia) from the Upper Triassic (Norian) of New Mexico increases the diversity and temporal range of the clade
Source: PeerJ. 2016 Sep 6;4:e2336. doi: 10.7717/peerj.2336 (PMC5018681; doi:10.7717/peerj.2336)
Supplement: Supplemental Information 1 — Selected measurements in cm unless otherwise specified. [file peerj-04-2336-s001.docx]

**Table 1**. Selected measurements (in cm unless otherwise specified) for *Vivaron haydeni*

| Measurement | (cm) | (cm) |  |
| --- | --- | --- | --- |
|  | **GR 263** | **GR 168** |  |
| Anteroposterior length of maxilla | 23.0 | 23.0 |  |
| Dorsoventral height of maxilla | 15.0 | - |  |
| Tooth serrations | 3/mm | 3/mm |  |
|  |  |  |  |
| Anteroposterior length of premaxilla not including dorsal processes | 5.5 |  |  |
| Anteroposterior length of premaxilla including dorsal processes | 8.5 |  |  |
|  |  |  |  |
|  | **Long axis** | **Short axis** | |
| Radius of most anterior premaxillary alveolus | 0.85 | 0.30 |  |
| Radius of second most anterior premaxillary alveolus | 1.1 | 0.65 |  |
| Radius of middle premaxillary alveolus | 1.3 | 0.60 |  |
| Radius of second most posterior premaxillary alveolus | 0.80 | 0.45 |  |
| Radius of most posterior premaxillary alveolus | 0.40 | 0.40 |  |
|  |  |  |  |
| Dorsoventral height of quadrate | 15 |  |  |
| Maximum length across quadrate ventral articular surface | 5.5 |  |  |
| Maximum length across quadrate dorsal articular surface | 1.5 |  |  |
| Depth of anteromedially opening fossa on quadrate | 1.6 |  |  |
|  |  |  |  |
|  | **GR 640** | **GR 451** |  |
| Anteroposterior length of ectopterygoid | 9.0 | 2.5 |  |
| Maximum length across ectopterygoid heads | 2.5 | 1.2 |  |
|  |  |  |  |
|  | **GR 560** | **GR 664** |  |
| Tooth serrations | 3/mm | 3/mm |  |
|  |  |  |  |
|  | **GR 638** | **GR 642** |  |
| Anteroposterior length of ilium | 22.0 | 18.0 |  |
| Dorsoventral height of acetabulum of ilium | 5.5 | 5.0 |  |
| Maximum mediolateral thickness of supraacetabular ridge of ilium | 3.0 | 2.5 |  |
| Anteroposterial length of supraacetabular ridge of ilium | 2.0 | 1.5 |  |
